# Olive Oil in the Mediterranean Diet and Its Biochemical and Molecular Effects on Cardiovascular Health through an Analysis of Genetics and Epigenetics

**DOI:** 10.3390/ijms232416002

**Published:** 2022-12-15

**Authors:** Renata Riolo, Riccardo De Rosa, Irene Simonetta, Antonino Tuttolomondo

**Affiliations:** U.O.C. di Medicina Interna Con Stroke Care, Promozione della Salute, Materno-Infantile, Di Medicina Interna e Specialistica di Eccellenza “G. D’Alessandro” (ProMISE), Università degli Studi di Palermo (Italy), Piazza delle Cliniche n.2, 90127 Palermo, Italy

**Keywords:** Mediterranean diet, olive oil, cardiovascular effects, genetics and epigenetics

## Abstract

Human nutrition is a relatively new science based on biochemistry and the effects of food constituents. Ancient medicine considered many foods as remedies for physical performance or the treatment of diseases and, since ancient times, especially Greek, Asian and pre-Christian cultures similarly thought that they had beneficial effects on health, while others believed some foods were capable of causing illness. Hippocrates described the food as a form of medicine and stated that a balanced diet could help individuals stay healthy. Understanding molecular nutrition, the interaction between nutrients and DNA, and obtaining specific biomarkers could help formulate a diet in which food is not only a food but also a drug. Therefore, this study aims to analyze the role of the Mediterranean diet and olive oil on cardiovascular risk and to identify their influence from the genetic and epigenetic point of view to understand their possible protective effects.

## 1. General Aspects of the Mediterranean Diet

The Mediterranean Diet (TMD) is the best known and most studied dietary pattern globally. It is characterized by high consumption of legumes, vegetables, fish, nuts, fruits, grains, seeds, olive oil, medium–low use of alcohol (especially red wine) and sweets, and low use of sugary drinks, processed and red meat. Along with the diet, there is also the so-called “Mediterranean lifestyle”, responsible for protective health effects and includes adequate hydration, daily physical activity, and social interactions during meals [1].

The modern definition of the Mediterranean diet includes many foods, such as tomatoes, potatoes, and beans, brought to the Mediterranean after Christopher Columbus discovered the “New World” [2].

TMD (typical in Greece and southern Italy), as the foundational diet for cardiovascular prevention, has the following characteristics:

- World’s highest life expectancy;
- Reduction in coronary heart disease rates, some types of cancer, and other chronic diseases related to nutrition [3,4];
- Dietary patterns associated with low chronic disease rates and high adult life expectancy [5,6];
- Based on olive oil, a fundamental constituent of TMD and beneficial for human health as it promotes the consumption of large quantities of vegetables in salads and equally significant amounts of legumes in cooked foods;
- Other crucial components of TMD are wheat, olives, grapes, and various derived products.

As a result, total lipid intake in this dietary model can be high (approximately or above 40% of total energy intake) in Greece or moderate (approximately 30% of total energy intake) in Italy.

Several observational studies have shown that adherence to TMD is linked to a better quality of life, greater longevity, and reduced mortality and morbidity from cardiovascular diseases, cancer, and other diet-related disorders [6,7,8,9,10]. Its intake has been correlated to a reduction in new cardiovascular events and a reduction in death after four years in patients with a previous cardiovascular event.

**Figure 1:**
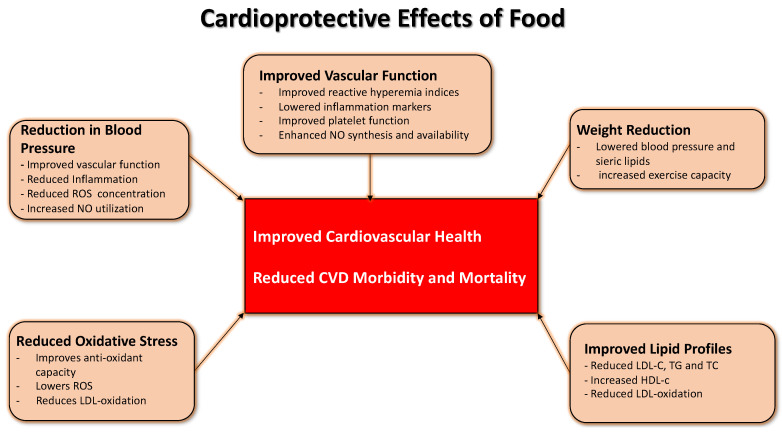
Proposed collective mechanisms to underpin the protective effect of foods against cardiovascular disease. No single ingredient or agent can account for specific food groups’ benefits. It takes a calorically sensitive and balanced variety to get the maximum help for the patients.

Over the years, prospective studies have been conducted that have analyzed the role of TMD in cardiovascular diseases. They have shown mainly favorable effects in the secondary prevention of cardiovascular diseases [8,11,12,13,14,15]; it can control hypercholesterolemia, hypertension [16], lipid metabolism, blood pressure levels, body mass index, and glycemic values [17] (Figure 1).

The 2015–2020 dietary guidelines (The 2015–2020 Dietary Guidelines for Americans) [16] consider TMD the primary strategy for preventing CVD, responsible for one-third of deaths globally.

**Figure 2:**
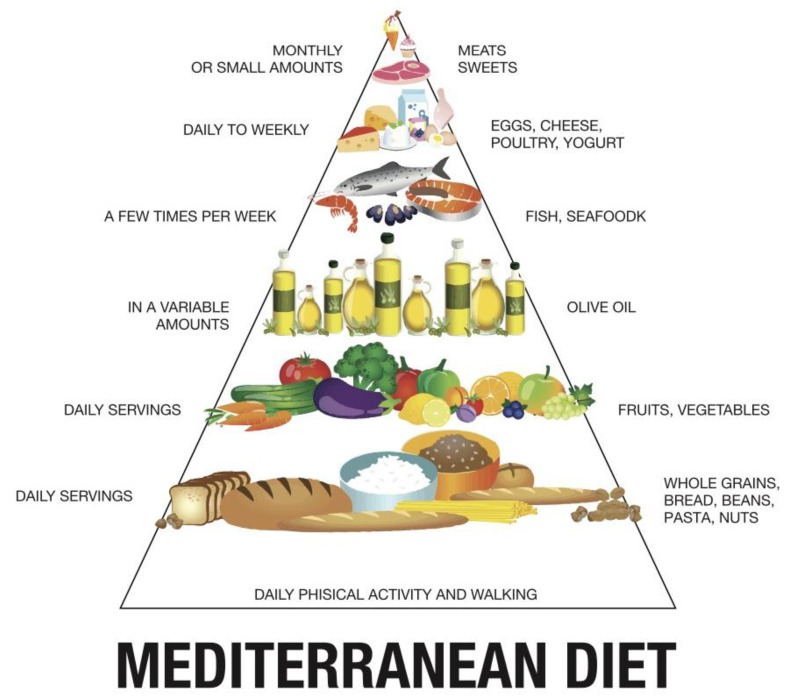
Image that represents the food pyramid typical of the Mediterranean diet. TMD is full of fruits and vegetables with an abundant consumption of olive oil. The pyramid describes, to varying degrees, which foods should be taken daily and which require monthly or infrequent intake.

Moreover, there are several ways to define a food pattern, including general descriptions, nutrition pyramids, a priori scoring systems, a posteriori food pattern formation, or based on food and nutrient content [6,18] (Figure 2).

By analyzing the foods commonly consumed by the population, a Mediterranean food model was created, represented in a triangle, with the base referring to the most frequently consumed foods and the upper part of the pyramid representing the most rarely consumed items (see Figure 2).

**Figure 3:**
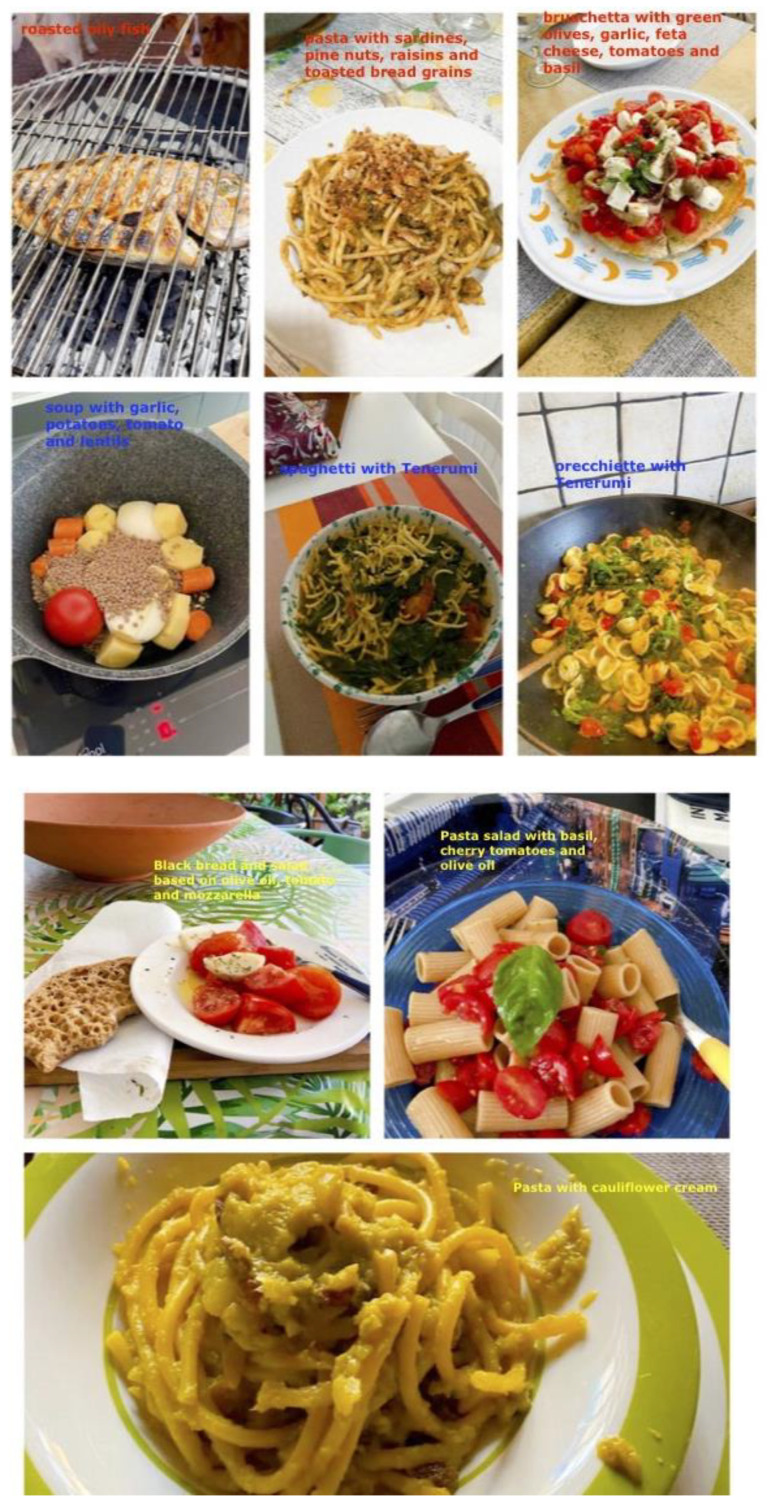
Some typical Mediterranean Italian food.

The remaining foods occupy intermediate positions. In particular, this model emphasizes the importance of the daily consumption of cereals and unrefined products (8 servings per day of whole grain bread, pasta, rice, etc.), vegetables (2–3 servings per day), fruit (4–6 servings per day), olive oil (the primary lipid added daily) and low-fat dairy products [19] (See Figure 3).

Coronary heart disease (CHD) represents a significant burden on global health. However, despite the well-known influence of dietary habits on the progression of this disease, there are no well-defined and scientifically valid nutritional approaches for the secondary prevention of CHD.

TMD is considered the diet with the lower risk of cardiovascular mortality, even though there is a high content of MUFA (Monounsaturated Fatty Acids) [20]. This importance comes mainly from the abundant consumption of olive oil, which is especially rich in MUFA and SFA (Saturated Fatty Acids) [21,22,23,24].

In Europe, more than 85 million people are currently (2015) living with cardiovascular disease, which causes nearly 4 million deaths per year, accounting for 45% of the total mortality burden. In addition, ischemic heart disease (IHD) and stroke mortality rates are generally higher in Central and Eastern Europe than in Northern, Southern, and Western Europe [25,26,27].

The excellent study PREDIMED [28] documented how TMD can reduce the risk of stroke compared with a low-fat diet (HR 0.60, 95% CI 0.45 to 0.80); however, it cannot reduce the incidence of cardiovascular or overall mortality. In contrast, in observational studies, TMD was associated with lower general and cardiovascular mortality [10,29,30].

High dietary saturated fat intake can increase blood concentrations of low-density lipoprotein cholesterol (LDL-C), an established risk factor for coronary heart disease. However, according to the Women’s Health Initiative, different foods appear to have other metabolic effects, which found no difference in cardiovascular disease in women who followed a low-fat diet for eight years.

PREDIMED study [22] enrolled 7447 Spanish participants at high CV risk (but without baseline CV disease) and was randomized into three groups: one group was given one Mediterranean diet enriched with extra virgin olive oil, the other a Mediterranean diet supplemented with mixed nuts, and finally one control group followed a low-fat diet for a median of 5 years. This sizeable Spanish cohort study reported that a Mediterranean diet supplemented with extra virgin olive oil or nuts significantly reduced the incidence of major cardiovascular events compared with a low-fat diet.

TMD with extra virgin olive oil group and TMD with nut group showed a significantly lower incidence of cardiovascular disease, a lower risk of stroke, myocardial infarction, CV death, and all-cause mortality compared with the low-fat diet [22].

Many nutritional studies to evaluate the protective role of TMD on the heart have used intermediate biomarkers (reduced blood pressure, cholesterol, inflammatory molecules, or other biomarkers) for cardiovascular risk analysis [31]. A sub-study of PREDIMED, focusing on lipoprotein particles, found that TMD has a role in increasing HDL molecules. In addition, this diet, enriched with walnuts, reduced LDL concentrations, documenting a protective role in atherogenesis. A recent report of the entire PREDIMED cohort, after a 4-year follow-up comparing the intake of TMD with the control diet, observed a reduction in diastolic blood pressure values [32].

Storniolo et al. have documented a reduction in serum ET-1 (Endothelin-1) levels in subjects with a Mediterranean nut diet. The decline in the concentration of ET-1 can determine the downregulation of the receptors responsible for the contractile effect of ET-1, such as ETAR (endothelin receptor A) and ETBR (endothelin receptor B), implicated in the pathogenesis of hypertension. In this way, the beneficial effect of TMD on blood pressure could be mediated, in part, through a reduced expression of ETAR and ETBR on muscle and endothelial cells, respectively, and the depletion of ET-1 levels [33].

Mind et al. identified a robust causal association between the development of cardiovascular disease and the intake of vegetables, nuts, monounsaturated fatty acids, and foods with a high glycemic index or fatty acids [5].

Nissensohn et al. demonstrated that TMD positively and significantly affects blood pressure (BP) in adults [34].

TMD has also shown a beneficial role in glycemic control. Concerning this, some studies showed that TMD, thanks to the production of short-chain fatty acids, can increase intestinal hormones GLP-1 and PYY, which inhibit gastric emptying, inducing satiety, and can control glycemic values in patients with type 2 diabetes mellitus [35,36,37].

Moreover, consumption of whole dairy products has long been considered a risk factor for cardiovascular health; these products contribute to the average daily intake of energy (11%), protein (14%), fat (17%), calcium (48%), phosphorus (24%), and vitamin A (27%). However, a recent meta-analysis has shown that the consumption of dairy products is not associated with CVD [38].

Some studies have proposed a distinction between dairy products and other dietary sources of SFAs based on their different effects on blood fats [39] and the possible cardioprotective effect of the consumption of fermented dairy products [40]. Goat’s milk, especially, possesses some intrinsic properties and an excellent nutritional quality determined by its lipid composition, making it an attractive alternative for developing high-value-added dairy products.

According to studies, the consumption of goat cheese naturally enriched with n-3 PUFA and conjugated linolenic acid (CLA) could potentially be a food rich in nutrients to improve health and act on cardiovascular prevention, as well as Halloumi cheese, the prominent Cypriot cheese [41,42,43]. Studies [44,45,46,47] show that the beneficial effects of goat cheese, above all, depend on organic farming practices that can positively influence the FA profile of the milk of all species, reducing SFA and increasing individual FA. Therefore, depending on the processing, the content of fatty acids varies, as does the ability to act on oxidative stress. Consequently, it is important not to demonize dairy products since scientific works have extensively analyzed their lipid composition and emphasized their benefits against oxidative stress.

## 2. Olive Oil: Types of Olive Oil and Its Constituent Elements

Olive oil is the primary source of fat in the Mediterranean diet.

According to the production mechanism, there are two types of olive oil, extra virgin olive oil (EVOO) and refined olive oil (ROO). EVOO is made directly from olives, the fruit of Olea Europea, by mechanical extraction, which consists of crushing and pressing the olives. On the other hand, ROO is subject to chemical extraction mechanisms, such as refining, which eliminates most of the minor components [48].

The derived benefits of olive oil consumption are mainly related to its high MUFA content. However, not many studies have been conducted on the possible role of minor components and the difference between EVOO and ROO.

The characteristics of the components of olive oil are closely linked to the degree of ripeness of the fruit and the location of growth (olives from colder places seem to have a higher content of MUFA [49].

**Figure 4:**
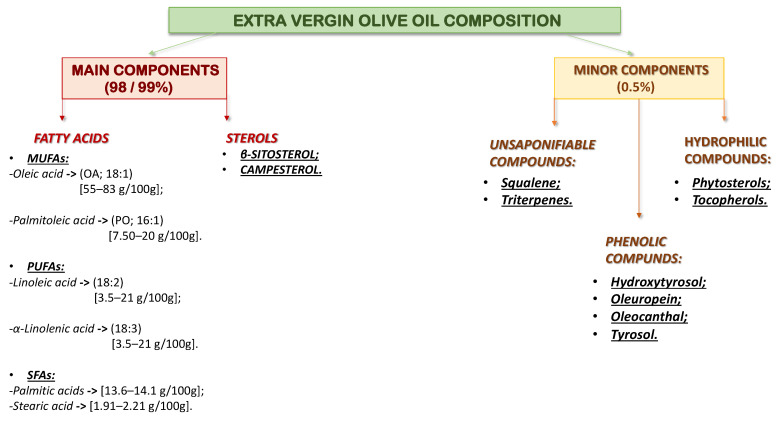
Extra virgin olive oil composition.

Olive oil contains the glyceric fraction, which makes up 99% of its composition, and the non-glyceric fraction (0.5%) (Figure 4) [50]. Among the MUFA components, oleic acid is the most represented (70–80% of the total), responsible for many positive health effects, such as a reduction in CVD, neurodegenerative diseases, and cancer [51].

A lower expressed fraction of PUFA (4–20%) is represented by linoleic acid and α-linolenic acid and a low concentration of SFA (stearic and palmitic acids) [52] (Figure 4). The lowest level of free fats in olive oil reduces the risk of developing inflammation. In addition, fatty acids increase cell apoptosis and generate insulin resistance [53].

However, some minor constituents include unsaponifiable compounds (triterpenes, squalene, pigments, etc.) and hydrophilic compounds (phenolic compounds, phytosterols, tocopherol, etc.) These minor components are the stars of EVOO because of their oxidative stability, sensory attributes (bitterness and spiciness), and unique fragrance [54].

Analyzing the components of EVOO, oleic acid (OA; 18:1n-9) and palmitoleic acid (PO; 16:1n-7) are the main MUFAs (Figure 4). These are natural acids in the diet but can also be synthesized from SFA derived from lipogenesis [55]. Because of the mentioned characteristics, foods enriched with MUFAs are highly recommended to reduce the risk of cardiovascular disease, reduce body weight, and produce other health benefits. In addition, it is possible that MUFA also have a protective role against drug-induced hepatotoxicity [55], reduce proliferation and apoptosis in smooth vascular muscle cells (CMVF), and enhance endothelial dysfunction and inflammation. These effects may be related to c-Jun N-terminal kinases (JNK-1/2) and nuclear factor kappa-light-chain-enhancer of activated B cells (NF-κB) pathways [56].

Polyphenols are other constituents of EVOO [51]. They are divided into flavonoids, phenolic acids (vanillic, cumastic, caffeic, protected, p-hydroxybenzoic, ferulic), flavones (apigenin, luteolin), lignans (acetoxypinoresinol, pinoresinol), phenolic alcohols (tyrosol, hydroxytyrosol), flavone glycosides (luteolin-7-O-glucoside, apigenin-7-O-glucoside), and secoiridoids (oleoresin, oleocantaline, oleuropein, p-HPEA-EA) [57]. They can reduce oxidative stress processes and possess cardioprotective, anti-diabetic, anti-obesity, anti-inflammatory cancer, neuroprotective, and various other effects [58]. The phenolic concentration in EVOO ranges from 50 to 800 mg/kg [59]. The most critical polyphenols in olive oil are oleuropein, hydroxybutyl (its hydrolytic degradation products), and tyrosol [59].

## 3. Effects of Olive Oil on Cardiovascular Risk

Olive oil contains monounsaturated fats, various antioxidant phenols, and other micronutrients that mediate the protective effects of CV through improvements in oxidative stress, endothelial dysfunction, inflammation, thrombosis, blood pressure, and metabolism of lipids and carbohydrates. The consumption of olive oil, especially extra virgin olive oil rich in phenolic antioxidants, seems helpful in preventing coronary heart disease (CHD). In 2011, the European Food Security Authority released an introductory statement for food safety (EFSA) based on several scientific research studies on the role of phenols in human health and their antioxidant effect [60].

The cardio-protection induced by olive oil has also been demonstrated by the results of a very recent analysis of two extensive prospective US studies, which showed that an increased intake of olive oil lowers the risk of morbidity and mortality of CV after twenty-four years of follow-up and that the replacement of dairy fat, margarine, butter, or mayonnaise with the equivalent amount of olive oil can significantly lower the risk of CV [61,62,63,64,65,66].

The Coronary Diet Intervention with olive oil and cardiovascular prevention (CORDIOPREV study) is an ongoing study to document how a TMD rich in extra virgin olive oil, compared to a low-fat diet, can influence the composite incidence of cardiovascular events after seven years in subjects with CHD. This study shows that olive oil intake is associated with a decrease in the progression of atherosclerosis, as demonstrated by a reduction in vascular intima and carotid plaque height. These results reinforce the clinical benefits of TMD in the context of secondary cardiovascular prevention [67].

The results of this study, therefore, once published, may significantly clarify the impact of olive oil intake on cardiovascular risk.

GREEK Acute Coronary Syndrome (GREEK), the prospective observational study, estimated, over ten years, the role of exclusive olive oil intake on the incidence of fatal and nonfatal cardiovascular disease in a sample of 2172 patients with acute coronary syndrome [68].

The study found that patients with ACS who consumed olive oil and added fats were significantly more likely to suffer a recurrent heart attack than those who used only olive oil [69].

This finding stressed the importance of exclusive olive oil intake in cardiopathic patients to reduce the risk of recurrent SCA events.

Meerpohl et al. also conducted a study comparing the various types of oil and evaluated direct and indirect tests, aimed at the classification of the different kinds of olive oil according to the effects on blood lipids and aimed at assessing their role in the risk of cardiovascular disease [70]. A mechanism of the action of nutrients concerns their ability to modulate gene and protein expression and, subsequently, the production of metabolites. Various meta-analyses have revealed that TMD has a protective effect on the action of different genes involved in vascular inflammation (in fact, TMD could affect changes in the overall transcriptomic response of genes related to cardiovascular risk [71]), both in the formation of foamy cells and in thrombotic phenomena [72].

Ferrè et al. conducted a study that included 61,181 women and 31,797 men who were free of heart disease, stroke, and cancer at baseline. Diet type was assessed using food intake frequency questionnaires at baseline and then every four years. The study documented that higher olive oil intake was associated with a lower risk of total CVD and CHD in two large prospective cohorts of US men and women. In addition, by analyzing the components, a diet free of margarine, butter, mayonnaise, and milk fat and, instead, rich in olive oil could reduce the risk of CHD and CVD [73].

Based on the EFSA opinion, European Community Regulation 432/2012 [74] reported the importance of olive oil polyphenols for health, as they interfere in oxidative stress mechanisms. An essential component of EVOO, Hydroxytyrosol (HT), seems to have anti-inflammatory effects. It can attenuate nitric oxide synthase (Inos), cyclooxygenase-2 (COX-2), tumor necrosis factor α (TNF-α), and Interleukin 1 (IL)-1β expression and can inhibit granulocyte and monocyte activation through the activity of hydroxybutol [75]; [76]. Another possible effect is related to interaction with microRNA, which regulates gene expressions in physiological and pathological contexts [77].

Oleuropein (another polyphenol in olive oil with potent antioxidant and anti-inflammatory activity) appears to reduce IL-1β expression [78]. It has shown a reduction in COX-2 and IL-17 expression in colon biopsies from patients with ulcerative colitis [79]. This polyphenol would seem to promote the reduction in nitric oxide expression in macrophages [80] and would play a hepatoprotective function through the partial induction of genes such as the transcription factor HO-1 and Nrf2, crucial for the cellular redox state [81].

Long-term and short-term studies have suggested that phenolic compounds are beneficial for the cardiovascular action of EVOO and have shown an improvement in antioxidant capacity [82,83], a reduction in F2-isoprostane, and production of ROS and serum sNOX2-dp [82,83,84,85], an activation marker of Nox2. In addition, integrated EVOO at a Mediterranean lunch is able to mitigate oxidative stress regulating endothelial dysfunction and platelet oxidative stress, as demonstrated by reduced Nox2 activity and the soluble release of E-selectina/VCAM1, respectively [84,85].

Molecules such as E-selectin and P-selectin, vascular cell adhesion molecule-1 (VCAM-1), and intercellular adhesion molecule-1 (ICAM-1) are predictors of endothelial dysfunction, representing an early marker of atherosclerosis events associated with cardiovascular risk factors.

Casas et al. [72] after 1 year of feeding with EVOO, showed a decrease in soluble adhesion molecules such as P-selectin, VCAM-1, and ICAM-1. In addition, measurements of increased flow-mediated dilation (FMD) [86], the internal thickness of the intima-media carotid artery, and plaque height [87] demonstrated the ability of the EVOO-supplemented medicinal product to improve endothelial function.

In contrast, Sala-Vila et al. [87] showed that EVOO integration showed no significant improvement in the conditions of the arterial wall.

Oxidized LDL (Ox-LDL) is a more powerful pro-atherosclerotic factor than native unmodified LDL. ROS are capable of initiating lipid peroxidation, resulting in the activation of total LDL and Ox-LDL formation [33].

Long-term studies (1 year) on subjects with high cardiovascular risk have documented a reduction in LDL values after the consumption of virgin olive oil compared to the control group [88].

In the EUROLIVE study, European participants were enrolled in a cross-study and randomly assigned to three olive oil groups, which were differentiated by their phenolic content (low, medium, and high) for three weeks. Following the intake of olive oil, the levels of oxidized LDL were reduced in a linear way with the increase in the phenolic content together with the changes in other oxidative markers. In addition, olive oil stimulates the synthesis of autoantibodies. OxLDL has a protective role in atherosclerosis [88], while no changes have been found in OxLDL and conjugated dienes [88].

The role of EVOO as anti-atherosclerotic nutrient is also supported by its ability to modulate human expressions of atherosclerosis-related genes in which LDL oxidation is involved. The intake of olive oil (25 mL/day) is also associated with a reduction in LDL oxidation and the expression of pro-atherogenic and pro-inflammatory genes linked to the CD40/CD40L pathway. This effect has led to a reduction in the surface area of its downstream products, such as vascular endothelial growth factor and monocyte chemoattractive protein-1, interleukin (IL)-8, and intercellular adhesion molecule-1 (ICAM1) via a decrease in mitogen activation of activated protein kinase-1 (MAPK1) [45]. The anti-inflammatory effect in the vascular wall can be another important mechanism that helps explain the link between EVOO and the development of cardiovascular disease.

Casas R et al. [88], in their recent study, showed improvement in people at high cardiovascular risk with long-term adherence to TMD supplemented with EVOO, suggesting a delay in the formation of atheroma plaque. In fact, they evaluated inflammatory markers such as high sensitivity C-reactive protein (hs-CRP), levels of major cytokines (IL-1, 5, 6, 7, 8, 12, TNF-α, and IFN), and levels of vulnerability plaque markers (IL-10, 13 and 18) [89].

The consumption of a Mediterranean-style diet supplemented with EVOO in patients with metabolic syndrome has been associated with a significant reduction in markers of systemic vascular inflammation (IL-6, IL-7, IL-18, and hs-CRP) [89].

These results, compared with long-term studies, may suggest that a more extended period of dietary intervention may be needed to change the concentration of specific markers.

With a significant decrease in inflammatory markers, such as thromboxane-B2 (TXB2) and leukotriene-B4, in long-term and short-term studies [82,83], the supplementation of EVOO confirmed the antithrombotic and anti-inflammatory effects of EVOO [82], [83].

Wang and Zhi [90] recently published a study based on a large sample, with which they evaluated the association between olive oil consumption and the risk of CVD and stroke. The authors documented that the consumption of olive oil at exactly between 20 and 30 g/day was associated with a lower risk of CVD and stroke. This extensive sample study provides significant evidence about the important role of olive oil to prevent and reduce the risk of CVD and stroke.

In light of this data, it is necessary that doctors should promote the intake of olive oil with the aim of acting on primary prevention and secondary prevention in order to minimize the risk of individual CVD [90].

## 4. Nutritional Genomics

Considering the role of olive oil on cardiovascular risk, it seemed interesting to evaluate whether olive oil could influence the anti-inflammatory processes from a genetic point of view.

On this subject, two disciplines were born, such as nutrigenetics and nutrigenomics, which make up nutritional genomics, with the aim of evaluating how foods can influence and regulate the activity of human genotype, both directly and indirectly, determining health status or disease [91].

Genetic analysis could be a tool to identify which individuals can benefit from a dietary intervention.

Little is currently known about gene–diet interactions, but as scientific research progresses, single nucleotide polymorphism (SNP) analysis from the entire genome is now acquired quickly and conveniently, increasing the prospect of creating personalized dietary recommendations based on the genetic variability of an individual with multiple SNP [92,93]. Studies of global human genomic variation have identified essential differences in the population regarding the allelic frequencies of common SNPs, which influence gene expression, regulating the metabolism of some of the most commonly consumed nutrients in humans. Through this analysis, it emerged that humans have adapted genetically to their ancestral diets and local environments, developing genetic differences that lead to a different response to a standard food route [94].

Whole Genome Association (GWAS) studies have identified many genetic variants with regards to nutrient absorption in pigs, lipid metabolism, the use of nutrients, and the accumulation of fat, which, in turn, are responsible for gene–diet interactions and human diseases [95].

Numerous DNA base pairs organized in chromosomes constitute the human genome. Genes occupy only a tiny fraction (<1%) of the genome; the rest include important regions to control the transcription of various genes, as well as repetitive regions and large regions with unknown functions. A single gene can produce multiple transcriptions each containing only specific exons, allowing individual genes to encode multiple protein isoforms. Although phenotypically different, humans generally differ by <1% [96].

Molecular biology has always been based on the concept that progression from DNA to RNA to protein is a simple process, along which multiple changes can occur, such as to alter the expression of genes and, in turn, impair the effect of any genetic variant. It should be borne in mind that individual variants may, however, not be expressed equally in all individuals. The Modern Western Diet (MWD) has documented how different genetic components and certain nutrients, when it comes to metabolism, have the ability to develop harmful gene–diet interactions. This process can alter molecular phenotypes (levels of bioactive nutrients and their metabolites) and clinical phenotypes, including human disease. A potentially dangerous gene–diet interaction [97] can be affected by different genetic, environmental, and biological components. First, other gene–diet interactions can result from the type of exposure to a human population. This exposure may vary if there are indeed genetic differences within an ethnic/racial population.

Another aspect related to gene–interaction is when a potentially harmful diet occurs when some individuals or ethnic/racial groups within a different population have a genetically different and metabolically varying ability to exploit a specific nutrient compared to others within the same group [98].

A further aspect to consider about gene–diet interactions concerns epigenetic alterations that affect crucial biological processes, for example, the metabolism of food nutrients. These epigenetic modifications are responsible for changes in gene expression and are often heritable, but unlike SNPs, they do not result in alteration in the DNA sequence. An example of this epigenetic alteration is DNA methylation within and around the promoter regions, which results in suppressed or reduced transcription of the gene and can be reversed or not methylated. These alterations are fundamental as they can compromise normal biological functioning but can also result from environmental exposure between diet and bioactive compounds [99].

DNA microarray probes (or “SNP chips”), albeit with some limitations, are the most common and economical form of genetic testing capable of rapidly detecting genotypes at hundreds of thousands of SNPs in the genome [100].

Considering the limitations of genetic analysis and the large number of SNPs detected, it would be useful to be able to identify which specific NSPs are able to influence genes in order to identify individuals and populations that may respond to specific dietary interventions. In light of this, some SNP genes specific to gene–diet interactions have been identified. For this purpose, we can refer as an example the gene FTO, whose alteration determines a greater caloric intake and an inhibition of satiety, which is why it is responsible for the development of obesity, resulting in increased cardiovascular risk and the onset of type 2 diabetes mellitus [101].

Apolipoprotein E (APOE), on the other hand, is fundamental for the maintenance of a correct concentration of plasma levels of cholsertolosis. Therefore, an alteration of the gene encoding this protein would increase the risk of cardiovascular disease [102].

Another possible genetic alteration, tx cxhat, which may increase the risk of cardiovascular disease, concerns the MTHFR gene, which regulates the activity of homocysteine. It has been shown that genotypes T/C and T/T at SNP rs1801133 [103] can increase levels of total homocysteine in the blood leading to hyperhomocysteinemia, a risk factor for a variety of medical conditions, especially teratogenic (neural tube defects, congenital and premature heart disease birth), pregnancy complications, tumors, adult cardiovascular disease, and neurodegenerative disorders. Supplementation of vitamin B 12 and folic acid may reduce plasma homocysteine levels but does not reduce the risk of heart disease in every instance with these genetic variants [104,105].

Considering that currently there are few studies related to the gene–diet interaction, Giovanell’s study has been interesting as it evaluates the functional variants in the renin-angiotensin (RAS) and kallikrein–kinin system genes (KKS) involved in the modulation of blood pressure (BP), verified effects of the interplay between genetic polymorphisms (rs699-AGT, rs4340-ACE, and rs1799722-BDKRB2) and the consumption of micronutrients (sodium, potassium, calcium, and magnesium), and the BP values of adult normotensive individuals. Biochemical, anthropometric, BP, and food intake data were evaluated for all participants. The results indicated that individuals with the G allele for rs699 polymorphism, in increasing sodium and magnesium consumption, both in the genotypic model and in the dominant model, had higher levels of systolic BP (SBP) than homozygotes. In addition, individuals with the T allele for rs1799722 polymorphism, with a rise in calcium intake, had significantly higher levels of SBP and diastolic BP (BPD) than homozygotes. Thus, these results indicated significant interactions between genetic polymorphisms (rs699-AGT and rs1799722-BDKRB2) and micronutrient consumption (sodium, magnesium, and calcium) on BP variation. These findings could contribute to an understanding of the complex mechanisms involved in the regulation of BP, which probably include several gene–nutrition interactions [106]

Many aspects of genetic testing are still limited, including the complexity of gene–nutrient interactions, the accuracy of genetic assessments, and the application of genetic knowledge; however, nutritional genomics could provide information on how diet and genotype interactions affect phenotype. Therefore, it is necessary for the healthcare professional to be trained in genetic counseling for the interpretation of data in order to identify the most appropriate nutritional approach, considering that individuals have different dietary needs and varied metabolism [107,108].

## 5. Olive Oil, Genes, and Cardiovascular Effect

Nocella et al. [109] conducted a study in which they demonstrated that EVOO appears to be relevant to reduce the incidence of cardiovascular events, including stroke and myocardial infarction. Chemically speaking, the main compound of EVOO is represented by fatty acids, in particular monounsaturated fatty acids, such as oleic acid. Tocopherols, polyphenols, and other minor constituents account for the remaining 1–2%. All these components can contribute to the “maintenance of health” with their beneficial effects of EVOO.

Guasch-Ferré et al. [110], in an observational study, found that the primary consumption of olive oil, in particular the EVOO variety, has been associated with a significant reduction in the risk of cardiovascular events and cardiovascular mortality in a Mediterranean population at high cardiovascular risk. They reported that increases of 10 g/d in EVOO intake were associated with a 10% reduction in the risk of cardiovascular events. Conversely, the consumption of common olive oil was not significantly associated with cardiovascular morbidity and mortality [110].

Previous nutrition-based studies have revealed that daily doses of olive oil in real life, as they are rich in fat and MUFA, have a protective effect on gene expression in relation to insulin sensitivity and the development of atherosclerosis in peripheral blood mononucleate cells (PBMC) [111,112].

The Castañer O et al. study documented how olive oil intake is associated with a dose-dependent downregulation of pro-atherosclerotic gene expression [71,113].

Olive oil can inflame atherosclerotic processes, which is why studies have shown a role in regulating the mechanisms of the molecules IL1β, IL1RN, TNF-α, and ICAM1.

The study of Camargo UN et al. [114] mainly documented a sub-regulation of IL1β. The gene coding for TNF-alpha was reduced in those who followed only the Mediterranean diet compared to those who followed a low-fat, carbohydrate-rich diet enriched in n-3 PUFA [114]. A reduced expression of ICAM1 has also been observed, a cell adhesion molecule and independent predictor of the development of type 2 diabetes and a crucial risk factor in cardiovascular mortality and morbidity [115].

Olive oil also contains a polyphenol, 2-(3,4-dihydroxyphenyl)-ethanol hydroxyphenyl, which can reduce vascular endothelial growth factor (VEGF) expression by blocking HIF-alpha activity (responsible for the formation and evolution of atherosclerotic plaque) [116].

Subjects who consume TMD and olive oil have documented a reduction in VEGF expression and NF-κβ expression, which acts in the signaling pathways of angiopoietin and hypoxia. NF-κβ alters signal transduction induced by hypoxia through the action of target genes encoding for interleukins and chemokines, such as IL1, IL8, and TNF-α, inflammatory enzymes, cell adhesion molecules, and inducible nitric oxide synthase [117]. Castaner et al. have also documented, in the group of patients who consumed olive oil rich in polyphenols, how the TMD, probably due to the altered regulation of the JUN gene, can affect both the processes related to hypoxia and the expression of nitric oxide, Enos, renin–angiotensin–aldosterone, P2Y, and cardiac hypertrophy signaling mechanisms [114].

The PREDIMED study documented how dietary components can determine a genetic predisposition for cardiovascular risk. This study emerged from the expression of the allele Ala in the sequence rs1801282 of the gene PPARγ2 (γ2 receptor activated by the peroxisome proliferator) in subjects following the Mediterranean regime and has documented beneficial effects for the presence of longer telomeres [118].

Ortega Azurim et al. have identified a possible correlation between the polymorphism rs3812316 and the gene MLXIPL (similar to a protein that interacts with MLX) documenting the beneficial effects of TMD against cardiovascular risk, especially for subjects who have followed TMD more consistently and who preset the allele G to the locus MLXIPL [119].

Observational and ex vivo studies have documented that long-term dietary adherence is responsible for the increased length of telomeres. According to Garcia Calzon et al., this aspect seems more evident in women who follow TMD and is not found in men. Some genetic variants can affect the length of the telomere following the approach of the Mediterranean diet. A slowdown in telomere shortening seems to be related to an improvement in the parameters of obesity and food inflammatory markers [120,121].

Garcia Ros recently studied the CLOCK gene, which is involved in glucose metabolism and with which he documented how a healthy lifestyle and diet can influence glucose metabolism positively. This effect appears to stem from the SNP rs1801260 in the CLOCK gene. Furthermore, the study by Lopez Guimera et al. also evaluated the correlation between expression of the CLOCK 3111T/C polymorphism and weight loss. In light of these results, genetic predisposition and increased exposure to TMD may influence several traits, such as glucose metabolism, lipid profile, and telomere length [122].

The study of Corella et al. [123] documented the role of TMD enriched with EVOO in the risk of developing diabetes mellitus type 2, atherosclerosis, and cardiovascular pathologies in subjects carrying the polymorphism TCF7L2 -rs7903146 (C > T). The gene of transcription factor 7-like 2 (TCF7L2) is the locus most associated with type 2 diabetes. The polymorphism rs7903146 (C > T) is one of the most important genetic variants influencing the risk of type 2 diabetes and the authors of this work have documented the subjects carrying the aforementioned polymorphism and those who did not undergo TMD. They had a higher risk of developing type 2 diabetes mellitus and cardiovascular disease (especially stroke) than those who had followed TMD enriched with olive oil. In particular, the follow-up of approximately five years for patients treated with TMD showed a lower incidence of stroke compared to subjects who had the polymorphism rs7903146 (C > T) and they did not follow any diet, and, above all, documented a significant improvement in the values of fasting blood sugar in those who followed the TMD compared to control groups [123].

Cardiovascular risk is also linked to the blood concentration of cholesterol, especially HDL and LDL. HDL is the component of “good” cholesterol that has the task of removing excess cholesterol from macrophages, thus, playing an important role in anti-atherogenic. Lipid-poor HDLs and small HDLs regulate the discharge of cholesterol by binding the ABCA1 protein (through a cellular system that depends on the expression of the caveolae/CAV1 gene in immune cells, such as macrophages). Large HDLs exert this same process through ATP G1 binding and B1 receptor scavenging. The expression of these cholesterol carriers is regulated by key transcription factors in lipid metabolisms, such as LXR and PPAR. In this regard, Farras et al. [124] conducted a recent study that documented the important role of the cardiovascular risk of VOO. In fact, the consumption of VOO has determined the expression of LXR and gene activation through over-regulation of CYP27A1 (a monooxygenase member of the cytochrome P450 superfamily), the enzyme responsible for the transformation of cholesterol into oxidized sterols, essential activators of LXR; on the other hand, the over-regulation of RXRα provides the protein needed to construct the interaction of LXR–RXR (active complex responsible for the nuclear functions of LXR). This aspect is fundamental because it could explain the beneficial effect of oil on the role of HDL, thus, improving its cardioprotective activity [124].

The anti-inflammatory and antioxidant activity of HDL is also regulated by the enzyme paroxonase and arylesterase (PON1), a glycoprotein with hydrolytic activity, which is associated with HDL in circulation. The gene encoding this enzyme is a member of a gene family, which also includes PON2 and PON3, all grouped in tandem on the long arm of human chromosome 7 (q21.22). PON1, binding HDLs, mobilizes cholesterol from macrophages, thus, playing an essential role in atherosclerosis and the development of cardiovascular disease [125]. PON1 activity is genetically regulated, with SNPs showing a strong association with arylesterase and paraoxanase activities; however, various factors can intervene and modulate PON1 and HDL activity (e.g., dietary factors, lifestyle, statins, smoking, etc.). Rizzi et al. [126] conducted a study identifying 5 SNPs for PON1 (rs854549, rs854551, rs854552, rs854571, rs854572) and for each SNP, evaluated the association with a high antioxidant diet, and identified the genotype with a significant cardiovascular protective effect. The study showed that a diet containing anthocyanins and polyphenols (present in olive oil for example) can promote the expression of protective genotypes in four independent polymorphisms, increasing levels of HDL and, thus, enhancing cardiovascular protection [126]. Therefore, PON1 variants could represent new biomarkers useful for providing targeted dietary recommendations to individuals for health promotion and for the implementation of preventive medicine strategies.

## 6. Olive Oil and Epigenetics

Epigenetics represent reversible hereditary changes that occur without alteration of the DNA sequence able to regulate gene expression. The epigenetic mechanisms identified to date are DNA methylation, histone modifications (acetylation and methylation), and post-transcriptional gene regulation by non-coding microRNAs (mRNA) [104].

In this regard, it seems that the anti-cancer, anti-inflammatory, anti-aging, and neuroprotective activity of olive oil is related to these epigenetic mechanisms [127].

The careful regulation of the “epigenome” determines whether, when, and where a gene is silenced or expressed. When epigenetic mechanisms are altered, chronic diseases, especially cancer, develop. As these processes are reversible, it is exciting how they can be influenced by dietary and environmental factors. In fact, during the course of life, we can witness the epigenetic variation induced by nutrition [128].

With regard to olive oil, two studies, one in rats [129] and one in humans [130], showed that EVOO is capable of modulating miRNA and genes with anti-inflammatory properties.

Nanda et al. [129], in fact, documented that in rats treated with DMH, EVOO intake reduced mRNA expression of NF-κB transcription factor and its target VEGF and MMP-9 genes [129].

D’Amore S. et al. [130] showed that in humans, the intake of polyphenol-rich EVOO suppressed the expression of kinase 3, which is associated with the interleukin-1 receptor involved in the regulation of NF-signaling κB and IL-8, and over-regulated the anti-inflammatory miRNA-23b-3p.

The anti-inflammatory effect of oleuropein has been studied in vitro in a RAW264.7 murine macrophage cell line and in human granulocytes/monocytes [131]. These compounds at low concentrations have the ability to inhibit the PMA-induced activation of granulocytes and monocytes. In murine macrophages, oleuropein was shown to reuse LPS-induced nitrites and PGE production, while it was able to inhibit LPS-induced regulation of myrrh-146a and induce nuclear translocation of NRf2 ((derived from erythroid 2)-like 2) [131].

The study by Scoditti et al. documented how hydroxyglycol is able to counteract the inflammation of adipocytes induced by TNF-α [132]. This has prevented TNF-induced ROS production, over-regulation of MCP-1, CXCL-10, M-CSF, IL-1, VEGF, COX-2, and MMP-2 at both miRNA and protein levels in the adipocytes of Simpson–Golabi–Behmel syndrome, and activation of NF-κB [132]. It also prevented TNF induced over-regulation of groups Mirna-34 and Mirna-155, and regulated let-7c levels in cells and exosomes [132].

Other studies [77,133] have suggested that hydroxytyrosol myrrh could act on the regulation of genes related to oxidative stress, lipid metabolism, and other metabolic processes.

Administration of this compound increased triglyceride levels. In particular, the diet supplemented with hydroxychol led to an increase in the expression of miRNA-193a-5p in healthy human subjects. In a transcriptomic analysis of mice, two new potential target genes for hydroxyglycol, Fgf21 and Rora, were found [77,133]. Therefore, these studies suggest but do not demonstrate, that miRNA modulated by hydroxyglycol contributes to the regulation of genes involved in oxidative stress, lipid metabolism, and other metabolic processes [77,133].

Considering the damage that oxidative stress causes in mitochondrial DNA, Quile et al. studied rats undertaking diets containing different sources of fat such as VOO and sunflower oil (SO). They documented the DNA breaking doubles in animals fed with olive oil compared to rats fed sunflower oil [134].

Another study, analyzing two regions of the mitochondrial genome (the ND1 and ND4 genes), documented that dietary fat can also modulate and eventually reduce the frequency of mtDNA deletions. For example, the lower increase in the rate of mtDNA deletion during aging in mice consuming olive oil is attributed to the lower number of free radicals produced by virgin olive oil (VOO) [135].

Fabiani et al. measured the effect of phenolic olive oil derivatives on H_2_O_2_, which induced DNA damage in human PBMC and promyelocytic leukemia cells (HL-60) [136].

A phenol extract of the olive oil complex (OOPE) and the oil mill wastewater (WW-PE) phenol extract has demonstrated a highly protective activity against DNA damage in PBMC and HL-60 cells.

Valentino Costantino et el. conducted a study to evaluate whether the benefits associated with TMD and VOO consumption could have protective effects leading to genetic changes in atherogenesis. The study showed that following TMD reduced oxidative and inflammatory status related to the expression of INF-γ, Rho, and interleukin-7 receptor (IL7R) in peripheral blood mononuclear cells. Thus, the Mediterranean diet with associated olive oil consumption showed a reduction in the expression of many of the genes involved in the process of atherogenesis. Moreover, the study also documented that after three months of TMD and olive oil intake, the reduction in ADRB2 gene expression is determined by the decrease in oxidative stress processes. These data are essential because they derive from the analysis of the effects of the Mediterranean diet and olive oil following their chronic intake [72].

According to some studies, olive oil may be able to induce epigenetic changes using its MUFA content. However, an in vitro study with human colon cancer cells (Caco-2) reported similar effects for VOO and its phenolic compounds [137]. In addition, some phenolic compounds may also affect DNA acetylation processes. For example, the secoiridoids (the oleuropein and its conjugated forms) can induce histone H3 hyperacetylation in cell cultures, among many other effects [138].

Some intervention studies have indicated that diets based on olive oil or the Mediterranean influence the methylation of DNA in genes coding for enzymes involved in the metabolism of fatty acids [139].

Aging can also be associated with changes in proteostasis (a mechanism that modulates the stability of proteomes in cells), such as pathologies occurrence. This aspect has been documented for Alzheimer’s and Parkinson’s disease and cataracts, where regular expression of unfolded, poorly folded, or aggregated proteins contribute to their onset [140]. Various in vitro studies have shown a beneficial role of olive phenolic compounds on protein aggregates. For example, through in vitro tests, oleuropein and oleuropein aglycone were documented to block the fibrillation process of the tau protein, which is typical of Alzheimer’s disease (AD) [141].

Rigacci et al. have also documented the beneficial role of oleuropein in patients suffering from type 2 diabetes mellitus. Usually, in diabetic patients, there is an accumulation in the pancreas of amylin, which is cytotoxic for the pancreas cells. Oleuropein aglycone, a derivative of olive oil, appears to interfere with the aggregation of amyl, inhibiting cytotoxicity. In addition, it also can influence cellular alteration. These crucial aspects suggest that aglycone oleuropein compromises amyloid aggregation and the formation of toxic prefibrillar infiltrates (oleuropein aglycon prevents cytotoxic accumulation of human amyloid) [142].

Continuous treatment with oleuropein has been shown to increase the degradation rates of proteasome derivatives in human embryonic fibroblast cultures. This process could result from conformational changes in the proteasome with a decrease in the number of oxidized proteins.

In addition, oleuropein-treated cells maintained proteasome function during replicative senescence. Cultures showed a delay in the onset of senescence morphology and an extension of mean lifespan by approximately 15% [143].

Bullion P. et al. also documented that feeding with EVOO can increase the mRNA levels of the L3 autophagy marker in older rats [84,144]. All these studies are fundamental as they document the importance of olive oil in protecting against protein damage.

Another critical aspect of the role of olive oil is the possible control of autophagy. Harrison DE et al. documented the role of rapamycin in mTOR. It has been found that through the constant or intermittent administration of the mTOR inhibitor, rapamycin can increase the lifespan of middle-aged mice [145] and may delay the effects of aging [145].

## 7. Microrna and Mediterranean Diet

TMD, therefore, could be involved in the regulation of several miRNAs related to metabolic syndrome (MS).

These small RNA molecules have been proposed as a new means of communication between different metabolic tissues, fatty acids, vitamins, and compounds found in fruits and vegetables, as they may be among the main elements capable of influencing their expression.

A recent study has revealed how the Mediterranean diet can induce changes in miRNA expression levels [146] and could be used as a potential therapeutic target for obesity and metabolic disorders [147].

The study of María I. Fontalba-Romero et al. [148] assessed the association between higher levels of Mir-590 and adherence to TMD with lower fat consumption. The study found that higher levels of Mir-590 were associated with a concentration of HDL in the normal range. The expression Mir-590, therefore, seems to be associated with the type and quantity of specific foods consumed, particularly foods such as fruit, commercial sweets, sugary drinks, and red or white meats. These results suggest that higher levels of miRNA represent healthy eating habits with a better metabolic profile. The Fontalba study also documented that Mir-192 inhibits the expression of genes involved in lipid metabolism, such as the very low density lipoprotein receptor (VLDLR) [149].

Other studies have documented how changes in Mir-590 concentration levels are associated with diabetes mellitus 2 in patients with blood stasis and many cardiovascular diseases, such as hypertension and type 2 diabetes mellitus [150].

Another study showed that Mir-590 could inhibit the activity of lactate dehydrogenase A [151].

MicroRNAs are also fundamental molecules for regulating cellular apoptosis and lipid metabolism. In addition, inhibiting target genes and blocking messenger RNA activity can also regulate cellular differentiation and glucose metabolism.

Yu-Jin Kwon et al. conducted a study that documented how eight weeks of TMD could cause changes in miRNA expression in breast cancer survivors with high BMI.

Chronic inflammation, insulin resistance, and obesity are modifiable risk factors that can also contribute to the development of breast cancer in women [152,153].

Given that breast cancer and CVD share common risk factors, the risk of cardiovascular disease is increased in older breast cancer survivors.

Weight control, exercise, and healthy eating could help reduce the risk of breast cancer recurrence and protect against other chronic diseases, including cardiovascular disease.

The study by Yu-Jin Kwon et al. documented how women who were breast cancer survivors and followed the Mediterranean diet had a reduction in cardiovascular events [85].

**Figure 5:**
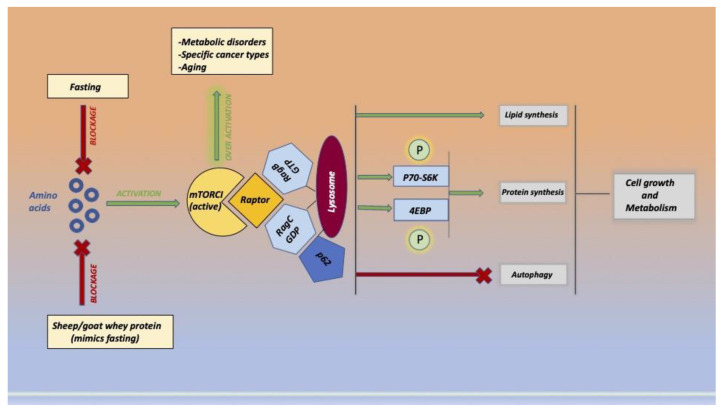
The image represents the molecular pathway of related amino acid activation of the mammalian target of rapamycin complex 1 (mTORC1) and the beneficial action of sheep/goat whey protein administration that imitates the fast. Mtor regulates different cellular processes, such as cell growth and proliferation, autophagy, and protein synthesis, and exists in two forms, mTORC1 and mTORC2.

Two mTORC1 (Figure 5) substrates are P70-S6 kinase 1 (P70-S6K1) and eukaryotic initiation factor 4E-binding protein 1 (4EBP1). When phosphorylated, mrn translation and protein synthesis are improved. The adapter P62 is an essential signaling molecule for mTORC1 physiological function since it interacts with S6K1 and 4EBP1. Whey protein, similar to fasting, inhibits the overactivation of mtor and exerts a potential protective role against various pathologies.

## 8. Action of Fatty Acids

The consumption of fatty acids, such as n-3 PUFA (EPA and DHA) and MUFA (oleic acid and palmitoleic), has been associated with an improvement in metabolic processes. Fatty acids, such as n-6 PUFA, saturated fatty acids (stearic and palmitic), and trans fatty acids (elaidic), are not only associated with the presence or development of obesity, T2D, pro-inflammatory profile, atherosclerosis, and IR, but can also act at the epigenetic level. Thus, they can regulate gene expression by modifying epigenetic mechanisms and consequently have a positive or negative impact on metabolic outcomes [154,155].

In recent years, there has been a change in AF intake, from diets high in MUFA and PUFA, to a Westernized dietary model characterized by a high content of saturated fatty acids (SFA) and trans fatty acids (TFA) and low in n-3 PUFA. This nutritional change is associated with an increasing prevalence of chronic non-communicable diseases (NCCDs) recently associated with aberrant and ongoing epigenetic changes, the leading cause of death worldwide [156].

**Figure 6:**
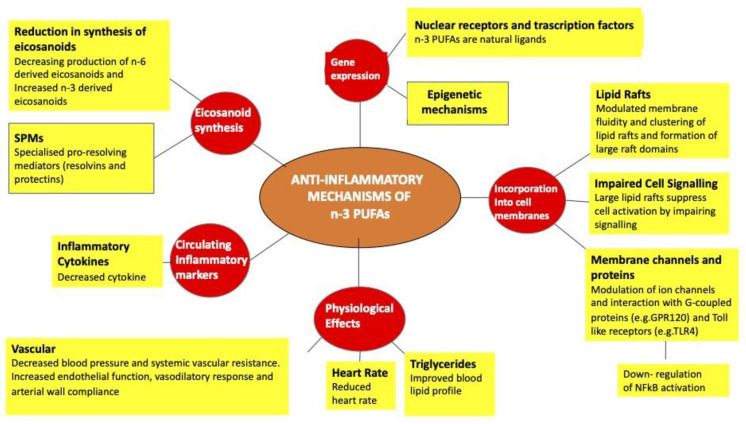
Description of the anti-inflammatory effects of fatty acids.

Tremblay BL et al. conducted a study that demonstrated how the addition of n-3 PUFA in the diet of overweight and obese subjects was associated with a different methylation pattern of 308 cpg sites (231 genes). In fact, of these, 286 cpg sites were hypermethylated, accounting for 93% of changes after integration and 22 were hypomethylated. These changes have determined an important effect by influencing the inflammatory and immune processes, lipid metabolism, T2D, and cardiovascular processes [157] (Figure 6).

Arpone et al. studied the effect of the TMD supplemented with EVOO or nuts on DNA methylation in the PREDIMED study (prevención con dieta mediterránea), and compared TMD + EVOO and TMD + nut diets with a low-fat control group over a five-year period and found that TMD + nuts manage to determine a cg01081346 hypermethylation in CPT1B/CHKB-CPT1B genes (carnitine palmitoyltransferase 1B/choline kinase-like, carnitine palmitoyltransferase 1B) and TMD + EVOO instead determine a hypomethylation in cg17071192 genes GNAS/GNASAS GNAS/GNASAS (Guanine Nucleotide Binding Protein, G Protein). However, the study showed that both diets guaranteed an intermediate metabolism and improved the genes involved in inflammatory diseases and protected from the development of diabetes [158].

Desgagné et al., considering that trans fatty acid (TFA) foods are associated with an increased risk of metabolic diseases, have studied how industrial TFA can inflate the concentrations of myrrh transported by HDL and plasma levels of HDL-c. In fact, a diet rich in industrial TFA has been shown to alter the concentrations of 5 miRNA in purified HDLs and to act on the 13 miRNA transported by HDLs in the plasma myrrh pool. These miRNA modified for their actions have shown an important role in the regulation of plasma lipid metabolism [159].

## 9. Conclusions

TMD is an important heritage that has been a source of exchange between cultures for thousands of years because of its wealth of fruits, vegetables, bread, fish, olive oil, poultry, and relatively low consumption of red meat and moderate wine during meals.

Attention to the type of nutrition should be paid daily, even within hospital facilities. During hospitalization, care should be taken to ensure the patient’s nutrition and to promote adequate resources and food quality management.

Cardiovascular diseases, such as heart attacks and strokes, are the leading cause of morbidity and mortality worldwide. This situation, especially in Italy, represents a fundamental problem of health expenditure, considering the progressive lengthening of the average age of the population and the multiple causes of hospitalization related to it.

The analysis has shown that the components of TMD have an important role in influencing the processes of oxidative stress, also regulating its mechanisms. Olive oil, thanks to its richness in polyphenols, has shown an important role in the prevention of hypertension, ischemic stroke, and myocardial infarction. Important is the growing role of olive oil both on the processes of DNA methylation and on the expression of messenger RNA, therefore, directly regulating the mechanisms of inflammation and oxidative stress, which are the basis of cardiovascular diseases.

However, it is necessary to emphasize that in the literature there is little information about the ability of olive oil to influence the expression of genes responsible for the synthesis of enzymes that positively or negatively regulate oxidative stress. There are also few studies that evaluate how olive oil can influence the expression of myrrh for the regulation of cardiovascular risk.

In view of the beneficial effects of olive oil, it is necessary to educate the entire population on foods that can positively contribute to cardiovascular prevention to ensure a healthy lifestyle. It would also be necessary to try to develop studies on a large population with a common diet to better understand the genetic changes induced by olive oil to exploit its protective action and eventually discover a therapeutic action.

## Data Availability

Not applicable.

## Abbreviations

CHD	coronary heart disease
TMD	Mediterranean diet
MUFA	monounsaturated fatty acids
SFA	saturated fatty acids
CAD	coronary artery disease
CVD	cardiovascular disease
VOO	virgin olive oil
ROO	refined olive oil
EVOO	extra virgin olive oil
PUFAs	polyunsaturated fatty acids
OO	olive oil
OA	oleic acid
PO	palmitate acid
JNK-1/2	c-Jun N-terminal kinases
NF-κB	nuclear factor kappa-light-chain-enhancer of activated B cells
VSMCs	vascular smooth muscle cells
HT	hydroxytyrosol
Nrf2	factor E2-related
COX-2	cyclooxygenase-2
CLA	conjugated linolenic acid
TNF-α	tumour necrosis factor α
VEGF	vascular endothelial growth factor
PBMCs	peripheral blood mononuclear cell
LDL-C	low-density lipoprotein cholesterol
GWAS	genome-wide association study
PPARγ2	γ2 receptor activated by the peroxisome proliferator
COQ10	coenzyme Q10
HMGCR	3-hydroxy-3-methylglyoxal-CoA reductase
ER	endoplasmic reticulum
VLDLR	deficient density lipoprotein receptor
ARHGAP15	GTPase activating protein15
ETBR	endothelin receptor B
ETAR	endothelin receptor A
